# Vitamin K epoxide reductase complex subunit 1 (*Vkorc1*) haplotype diversity in mouse priority strains

**DOI:** 10.1186/1756-0500-1-125

**Published:** 2008-12-01

**Authors:** Ying Song, Nicole Vera, Michael H Kohn

**Affiliations:** 1 Department of Ecology and Evolutionary Biology, Institute of Biotechnology & Bioengineering, Rice University, MS 170 205A Anderson Biology Lab 6100 Main Street, Houston, Texas 77005, USA; 2 University of Texas Southwest medical center, Dallas, Texas 75390, USA

## Abstract

**Background:**

Polymorphisms in the vitamin K-epoxide reductase complex subunit 1 gene, *Vkorc1*, could affect blood coagulation and other vitamin K-dependent proteins, such as osteocalcin (bone Gla protein, BGP). Here we sequenced the *Vkorc1 *gene in 40 mouse priority strains. We analyzed *Vkorc1 *haplotypes with respect to prothrombin time (PT) and bone mineral density and composition (BMD and BMC); phenotypes expected to be vitamin K-dependent and represented by data in the Mouse Phenome Database (MPD).

**Findings:**

In the commonly used laboratory strains of *Mus musculus domesticus *we identified only four haplotypes differing in the intron or 5' region sequence of the *Vkorc1*. Six haplotypes differing by coding and non-coding polymorphisms were identified in the other subspecies of *Mus*. We detected no significant association of *Vkorc1 *haplotypes with PT, BMD and BMC within each subspecies of *Mus*. *Vkorc1 *haplotype sequences divergence between subspecies was associated with PT, BMD and BMC.

**Conclusion:**

Phenotypic variation in PT, BMD and BMC within subspecies of *Mus*, while substantial, appears to be dominated by genetic variation in genes other than the *Vkorc1*. This was particularly evident for *M. m. domesticus*, where a single haplotype was observed in conjunction with virtually the entire range of PT, BMD and BMC values of all 5 subspecies of *Mus *included in this study. Differences in these phenotypes between subspecies also should not be attributed to *Vkorc1 *variants, but should be viewed as a result of genome wide genetic divergence.

## Background

The warfarin-sensitive vitamin K epoxide reductase enzyme complex (VKOR) converts vitamin K 2,3-epoxide to vitamin K hydroquinone, a required cofactor for the post-translational gamma-carboxylation of several blood coagulation factors and other vitamin K-dependent proteins, such as osteocalcin (bone *Gla *protein, BGP) and matrix *Gla *protein (MGP) [1,2]. MGP has also been found in blood vessel walls and in association with atherosclerotic plaques [3,4]. The vitamin K epoxide reductase complex subunit 1 gene (*Vkorc1*) appears to be a critical component of the VKOR [5,6].

The laboratory mouse is a widely used model organism to study human physiology and medical conditions. Moreover, the *Vkorc1 *has recently been discussed in the context of gene-drug interactions and disease. For example, genetic polymorphisms in the *Vkorc1 *could affect anticoagulant therapy with warfarin in humans [7,8]. Genetic variants in the *Vkorc1 *have been shown associated with genetically determined resistance to warfarin in rodents [6,9]. Genetic polymorphisms in the *Vkorc1 *gene may also affect other vitamin K-dependent reactions, such as bone mineralization and atherosclerotic calcification [10-12]. For example, low VKOR activity may result in lower levels of post-translational gamma-carboxylation of BGP, and, conceivably, reduce the BGP-dependent phenotypic measures bone mineral density (BMD) or bone mineral content (BMC). Osteoporosis-related fractures have been postulated side effects of anticoagulant treatment with warfarin, which inhibits the VKOR [12].

So-called mouse priority strains currently are subjected to intensive phenotyping surveys. The results of these are accessible via the Mouse Phenome Database (MPD) at the Jackson Laboratory, Bar Harbor, Maine [13]. If genetic variation in the *Vkorc1 *for the panel of mouse priority strains would be known then searches for possible associations of *Vkorc1 *haplotypes with simple and complex vitamin K-dependent phenotypic measurements could be initiated. However, so far only 5 *Vkorc1 *haplotypes have been identified in 16 strains representing 4 subspecies (c.f. MPD, Perlgen data; accessed September 2008).

This shortage of data prompted us to collect genetic polymorphism data for the *Vkorc1 *gene in mouse priority strains. In addition, we explored patterns of association between *Vkorc1 *haplotypes and the vitamin K-dependent phenotypes prothrombin time (PT), bone mineral density and content (BMD and BMC) currently available in the MPD.

## Methods

### Discovery of Vkorc1 haplotypes

**Figure 1:**
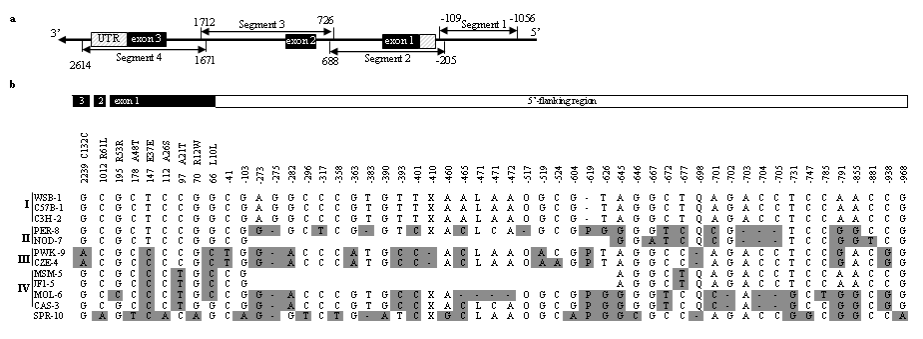
**a. Genomic organization of the mouse vitamin K epoxide reductase complex subunit 1 gene (*Vkorc1*) and sequencing strategy**. Exons are depicted in black and the untranslated regions (3'- and 5'-UTR) are shaded in gray. +1 indicates the transcription start. The primer pairs used to amplify segments 1–4 were (5' to 3') -1056seg1F **CAC GAC GTT GTA AAA CGA C**GG TCT GGG AAA TCA CAG GAA, -109seg1R **GGA TAA CAA TTT CAC ACA GG**C TAG GAA TGC TGG CGT GGT A, -205seg2F **CAC GAC GTT GTA AAA CGA C**TG CAG CCT CTC CAA CTA CAA T, 688seg2R **GGA TAA CAA TTT CAC ACA GG**A TGT GCC ACC TCA CAA ACA A, 726seg3F **CAC GAC GTT GTA AAA CGA C**CG TTC GGG AGT TGA GTC TCT, 1712seg3R **GGA TAA CAA TTT CAC ACA GG**A CCT ACC AGG TGT GGT CCA A, 1671seg4F **CAC GAC GTT GTA AAA CGA C**GT GCT GGG ATT AAA GCA TGG, 2614seg4R **GGA TAA CAA TTT CAC ACA GG**G AAA GAC TGA CAC CCC GAA G. The M13 primer tail used for sequencing is shown in bold face type. F and R refer to forward primer and reverse primer, respectively. b. Overview of polymorphisms in the coding and 5' region of the*Vkorc1*. For each polymorphism the location in the mouse reference sequence (Ensembl No. ENSMUSG00000030804) and whether these are synonymous or non-synonymous is indicated. Strains (represented by a three-letter code) representative for haplotypes 1–10 are shown. I-IV refer to the clades identified by phylogenetic analysis of *Vkorc1 *haplotypes (c.f. Figure 2b). Nucleotides different from those in the reference sequence are shaded in gray. -indicate insertions/deletions (indels) and blank spaces indicate ambiguous or missing sequence. Five multiple-base indels are replaced by X (CTGTAAAGCTACTATTAACAGGACGGTA), L (ACACA), O(ATTGGGT), P (CAGCCCC), and Q (AGA).

Forty mouse priority strains were obtained (the DNA resource at the Jackson Laboratory, Bar Harbor, Maine) [c.f. Additional file 1]. Polymerase Chain Reactions (PCR) were used to amplify the entire *Vkorc1 *gene and its 5' region (Figure 1a). PCR was performed with Go*Taq*^®^DNA polymerase (Promega, Madison, WI) in 1× buffer containing 1.5 mM Mgcl_2_. PCR thermocycling conditions were as follows: 2 minutes at 94°C, followed by 30 cycles of 30 seconds at 94°C, 30 seconds at 55°C, 1 minute at 72°C, and a final 5 minutes at 72°C. Products were cleaned using *Exo*SAP-IT (USB, Cleveland, OH), and sequenced in both directions on ABI Prism™ 3730xl DNA sequencers (Applied Biosystems, Foster City, CA).

Sequences were assembled, proofread and aligned to the published sequence of the *Vkorc1 *of the mouse (C57BL/6J) using LASERGENE 7.2 [14]. Single nucleotide polymorphisms (SNPs) and insertions/deletions (indels) were identified and verified by eye. Haplotypes were inferred by using ARLEQUIN 3.11 [15].

### Phylogenetic analysis of the Vkorc1 haplotypes

Subspecies of the mouse may differ in their phenotypes merely as a function of the genetic differences accrued across the genome during independent evolution. Thus it is important to limit direct tests for association of *Vkorc1 *haplotypes with phenotypic traits to groups of closely related strains, or clades, of the same subspecies of *Mus*. We used phylogenetic methods (ignoring recombination) to identify such clades based on the identified *Vkorc1 *haplotypes.

Specifically, we studied five subspecies of *Mus *represented by 31 inbred- and 9 wild-derived strains [see Additional file 1]. *M. m. domesticus *is the commonly used laboratory mouse. *M. m. musculus, M. m. spretus, M. m. castaneus *and *M. m. molossinus *are subspecies of *Mus *that have contributed to varying degrees to the gene pool of some *M. m. domesticus *strains. To reconstruct the evolutionary relationships between strains neighbor-joining (NJ) and maximum parsimony (MP) trees were built using the option of complete deletion and 1,000 bootstrap replicates as implemented in MEGA 4.0 [16]. Genetic distances underlying the NJ method were corrected by the Kimura 2-parameter model [17]. *M. m. spretus *(SPRET/EiJ) was used to root the tree [18,19].

### *In silico *association analyses based on the Mouse Phenome Database (MPD)

Phenotypic measurements PT [20], BMD [21] and BMC [21] for each strain were retrieved from the MPD [22]. The strain PWD/PhJ was excluded for lack of data. STATISTICA 8.0 [23] was used to identify clusters of strains based on each phenotypic measurement (transformed to the city-block/Manhattan linkage distance) by applying the unweighted pair-group method with arithmetic means (UPGMA). We tested for the association of *Vkorc1 *haplotypes with each phenotypic measurement within and between clades (as defined by phylogenetic analysis) using Mann-Whitney U tests.

## Results and discussion

### Vkorc1 genetic polymorphisms in mouse priority strains

We identified 10 haplotypes that were defined by 84 SNPs and 153 indels [see Additional file 1]. Six haplotypes were identified when the coding region was considered (Figure 1b). Five non-synonymous SNPs altering five amino acids (R12W, A21T, A26S, A48T and R61L) and four synonymous SNPs were identified (Figure 1b). The C132C polymorphism unique to *M. m. musculus *mapped onto the evolutionary conserved redox center C132-X-X-C135 motif of the *Vkorc1 *[24,25].

Consideration of SNPs and indels in the 5' region increased the number of haplotypes to nine (Figure 1b), and inclusion of intronic polymorphisms increased the number of haplotypes to ten. All *M. m. domesticus *strains were identical in the coding region but genetic polymorphisms in the 5' region separated *M. m. domesticus *into two groups (Figure 1b). However, our comprehensive survey uncovered only two previously known *Vkorc1 *haplotypes (1 and 7) and two novel haplotypes (2 and 8) in *M. m. domesticus*. Considering the coding and 5' regions, only synonymous SNPs and 5' polymorphisms distinguished *M. m. domesticus *and *M. m. musculus*. A single non-synonymous SNP distinguished *M. m. domesticus *and *M. m. musculus *from *M. m. molossiunus *and *M. m. castaneus*. Four non-synonymous SNPs and at least eight 5' variants were unique to *M. m. spretus*.

### Phylogenetic analysis of Vkorc1 haplotypes

**Figure 2:**
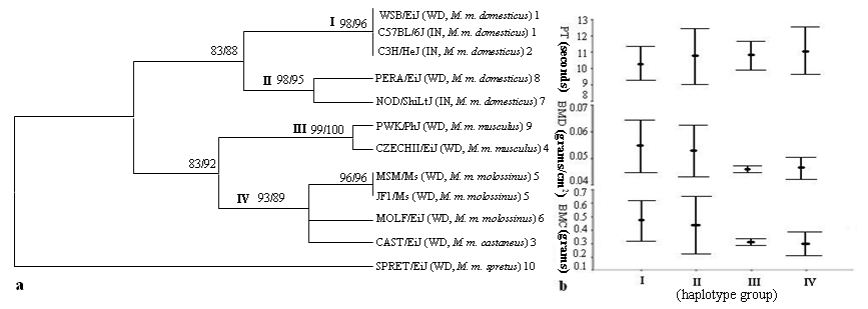
**a. Neighbor-joining (NJ) tree showing evolutionary relationships of priority strains as inferred based on the *Vkorc1 *and its 5' region**. Each strain is followed by the subspecies name and the haplotype group it belongs to [c.f. Additional file 1]. IN and WD indicate whether strains are inbred or wild derived, respectively. Too many inbred strains are represented by C57BL/6J since they share one haplotype. Numbers on the branch indicate the bootstrap values from NJ and MP analyses. b. The mean and standard variation of the phenotypes PT and BMD/C. The clades I-IV are shown on the X-axes and their corresponding PT, BMD and BMC measurements are shown on the Y-axes.

The analysis of *Vkorc1 *haplotypes using the NJ method resulted in four main clades that were supported by bootstrap values of 93% or higher (Figure 2a). All 30 inbred strains and one wild-derived *M. m. domesticus *strain fell into clade I. One inbred and one wild-derived *M. m. domesticus *strain together formed clade II. Wild-derived *M. m. musculus *strains formed clade III and wild-derived *M. m. molossinus *and *M. m. castaneus *strains formed clade IV. The overall topology of the *Vkorc1 *haplotype tree is consistent with those obtained during previous phylogenetic studies of mice [19,26]. It was also supported by bootstrap values of 89% or higher when MP was used to analyze the *Vkorc1 *haplotype data.

### Relationship between Vkorc1 polymorphisms and prothrombin time

Variation in PT within and between clades I-IV was substantial. However, variation in PT within *M. m. domesticus *(clades I and II) overlapped with PT variation in the other subspecies (Figure 2b). In particular, the mean (and standard deviation) of PT was 10.318 (0.514) for clade I, 10.772 (0.857) for clade II, and 10.345 (0.541) for clades (I + II) (i.e. within *M. m. domesticus*). The corresponding values for clades III and IV, respectively, were 10.850 (0.451) and 11.075 (0.729).

None of the clusters identified based on PT values for each strain was comprised out of groups of related *Vkorc1 *haplotypes as defined by the phylogenetic analysis [see Additional file 2]. Most notably, haplotype 1 was observed in conjunction with the entire range of PT values present in the data, and it clustered with a wide range of genetically different haplotypes from various distinct clades. For example, the strains CBA/J (haplotype 1) and JF1/Ms (haplotype 5), which differ by 5' polymorphisms as well as one non-synonymous and two synonymous polymorphisms, clustered together based on their PT values (Figure 1b). Similarly, A/J (haplotype 1) and PWK/PhJ (haplotype 9), which differ in at least 10 polymorphisms in the 5' region and 3 synonymous SNPs, clustered together.

Statistical analysis confirmed this suggested lack of association between PT and *Vkorc1 *haplotypes within each of the clades I-IV (Mann-Whitney U tests, all N.S. at α = 0.05). Moreover, no significant association was found between haplotypes identified in *M. m. domesticus *and PT (clades I vs. II) (P = 0.389), suggesting 5' polymorphisms have little if any effect. Even clades differing by a non-synonymous SNP (A21T) as well as 5' polymorphisms did not significantly differ in PT (clades III vs. IV) (P = 0.733). Thus, association between the vitamin K-dependent phenotype PT and haplotype variation of the *Vkorc1 *was not statistically supported within subspecies and between sister clades of subspecies as defined by the *Vkorc1 *haplotype tree.

However, the genetic distance between *Vkorc1 *haplotypes accrued between more divergent subspecies was correlated with differences in PT (clades I vs. III; P = 0.022) and (clades I vs. IV; P = 0.006). However, given the large number of mutations across the genome separating these clades, this association is, in our view, not an indication of an association between *Vkorc1 *haplotypes and variation in PT between clades.

### Relationship between Vkorc1 polymorphisms and bone mineralization

Similar to the results for PT variation in BMD within and between clades I-IV was substantial (Figure 2b), with the entire range of variation present in *M. m. domesticus *(clades 1+II). In particular, the mean (and standard deviation) of BMD was 0.055 (0.005) for clade I, 0.053 (0.005) for clade II, and 0.054 (0.005) for clades (I + II) (i.e. within *M. m. domesticus*). The corresponding values for clades III and IV, respectively, were 0.046 (0.001) and 0.046 (0.002). A similar pattern was observed for BMC (Figure 2b).

Analysis of BMD and BMC values resulted in two major clusters [c.f. Additional file 2]. One major cluster was comprised out of the *M. m. domesticus *haplotypes 1, 2, and 7. The other major cluster included a wide range of genetically different haplotypes from all subspecies of *Mus*, suggesting that there is no clear association of *Vkorc1 *haplotypes with BMD and BMC values. For instance, haplotype 1, which was found in conjunction with the entire range of BMD and BMC values, also clustered together with SPRET/EiJ (haplotype 10) based on BMD. However, these two haplotypes differ by as many as 4 non-synonymous SNPs and 21 polymorphisms in the 5' region (Figure 1b).

Statistical analysis confirmed this suggested lack of association between BMD and BMC and *Vkorc1 *haplotypes within each of the clades I-IV (Mann-Whitney U tests, all N.S. at α = 0.05). Moreover, no significant association was found between haplotypes identified in *M. m. domesticus *and BMD and BMC (clade I vs. II) (P = 0.510 for BMD and P = 0.582 for BMC) suggesting 5' polymorphisms have little if any effect on the phenotypes. Even clades differing by a non-synonymous SNP (A21T) as well as 5' polymorphisms did not significantly differ in BMD and BMC (clade III vs. IV) (P = 0.671 for BMD and P = 0.497 for BMC). Thus, within subspecies or between closely related subspecies no association between the vitamin K dependent phenotypes BMD and BMC and haplotype variation of the *Vkorc1 *was found.

However, when genetically more divergent clades were compared an association of *Vkorc1 *haplotypes with BMD and BMC was observed (clade I vs. III; P < 0.001 for BMD and P = 0.001 for BMC and clade I vs. IV; P < 0.001 for BMD and P = 0.001 for BMC). However, as discussed above for PT, this association should not be interpreted as direct evidence for the role of the *Vkorc1 *in determining BMD and BMC.

## Conclusion

The comprehensive SNP survey covering the *Vkorc1 *and its 5' flanking region of 40 mouse priority strains should be a valuable resource for future tests of an association between vitamin K-dependent phenotypes and *Vkorc1 *haplotypes. For example, the absence of *Vkorc1 *haplotype variation in *M. m. domesticus *in clade I enables a rapid assessment of the role of the *Vkorc1 *underlying variation in a phenotype. However, in this study, variation of PT, BMD and BMC could not be explained by *Vkorc1 *haplotype, as evidenced in particular by the range of phenotypic variation seen in conjunction with *M. m. domesticus *haplotype 1.

Contrasts between *M. m. domesticus *strains of clade I and II may be informative with regard the role of polymorphisms in the 5' region of the *Vkorc1*. In our study of PT, BMD and BMC, however, this 5' sequence variation of the *Vkorc1 *did not significantly explain the variation in these phenotypes. We note that only two strains currently belong to clade II, which precluded more powerful statistical tests for association. Additional strains belonging to this clade should be identified.

Overall, phenotypic variation in PT, BMD and BMC in *M. m. domesticus*, which is substantial, appears to be dominated by genetic variation in genes other than the *Vkorc1*. Phenotypic differences between subspecies should be interpreted as a byproduct of genome wide divergence. We suggest that it would be of interest to measure differences in warfarin tolerance between mouse priority strains, particular, between strains belonging to clades I and II, because warfarin tolerance in humans and warfarin resistance in rodents had been attributed to *Vkorc1 *haplotype variation. In addition, MGP is a vitamin K-dependent protein that is a potent inhibitor of the calcification of the cardiovascular system. Thus, it would be of interest to examine the degree to which the calcification of the cardiovascular system is associated with *Vkorc1 *haplotypes in mouse priority strains belonging to clades I and II. However, in the current panel of the common laboratory mouse priority strains of *M. m. domesticus *most variation in potentially vitamin K-dependent phenotypes appears to be due to variation in genes other than the *Vkorc1*.

## Competing interests

The authors declare that they have no competing interests.

## Authors' contributions

This work is part of the honors thesis of NV, who helped generating data. YS generated data, analyzed the data and wrote the paper. MHK conceived the study, helped analyzing data, and wrote the paper.

## Supplementary Material

Additional file 1 **List of haplotypes inferred from 237 polymorphisms for 40 mouse priority strains.** To be consistent with the *Vkorc1 *direction in the mouse genome, the polymorphisms (5'→3') start from right to left. Question marks and the blank indicate the gaps and missing data, respectively. Locations of polymorphisms in the mouse *Vkorc1 *reference sequence (Ensembl No. ENSMUSG00000030804) are shown on the top. SNPs identifiers (rs number) are shown for the published SNPs. Five non-synonymous polymorphisms are indicated with Arg12Trp, Ala21Thr, Ala26Ser, Ala48Thr and Arg61Leu, and four synonymous polymorphisms are indicated with Leu10Leu, Glu37Glu, Arg53Arg, and Cys132Cys. Negative numbers indicate the locations of the polymorphisms in the 5' flanking region. Seven multiple-base indels are replaced by E (ATGCCTTTGATCCCAGCACTTCTGAGGCAGAAGCAGGCAAAGCTCTGAGTTCAAGGCCAGCCTGGTCT), Z (ACACAGTTC), X (CTGTAAAGCTACTATTAACAGGACGGTA), L (ACACA), O(ATTGGGT), P (CAGCCCC), and Q (AGA). Color coding of SNPs as employed by the Mouse Phenome Database SNP reports . Click here for file

Additional file 2 **Dendrogram for 40 mouse priority strains with haplotypes 1–10 (haplotype identifiers in parentheses) based on the prothrombin time (PT) and bone mineralization (BMD/C) values.** Haplotypes different from those found in most of the commonly used strains are labeled in red. Click here for file
