# Supplementary figures and images for: Vitamin K epoxide reductase complex subunit 1 (Vkorc1) haplotype diversity in mouse priority strains
Source: BMC Res Notes. 2008 Dec 1;1:125. doi: 10.1186/1756-0500-1-125 (PMC2629770; doi:10.1186/1756-0500-1-125)

Dendrogram for 40 mouse strains based on PT

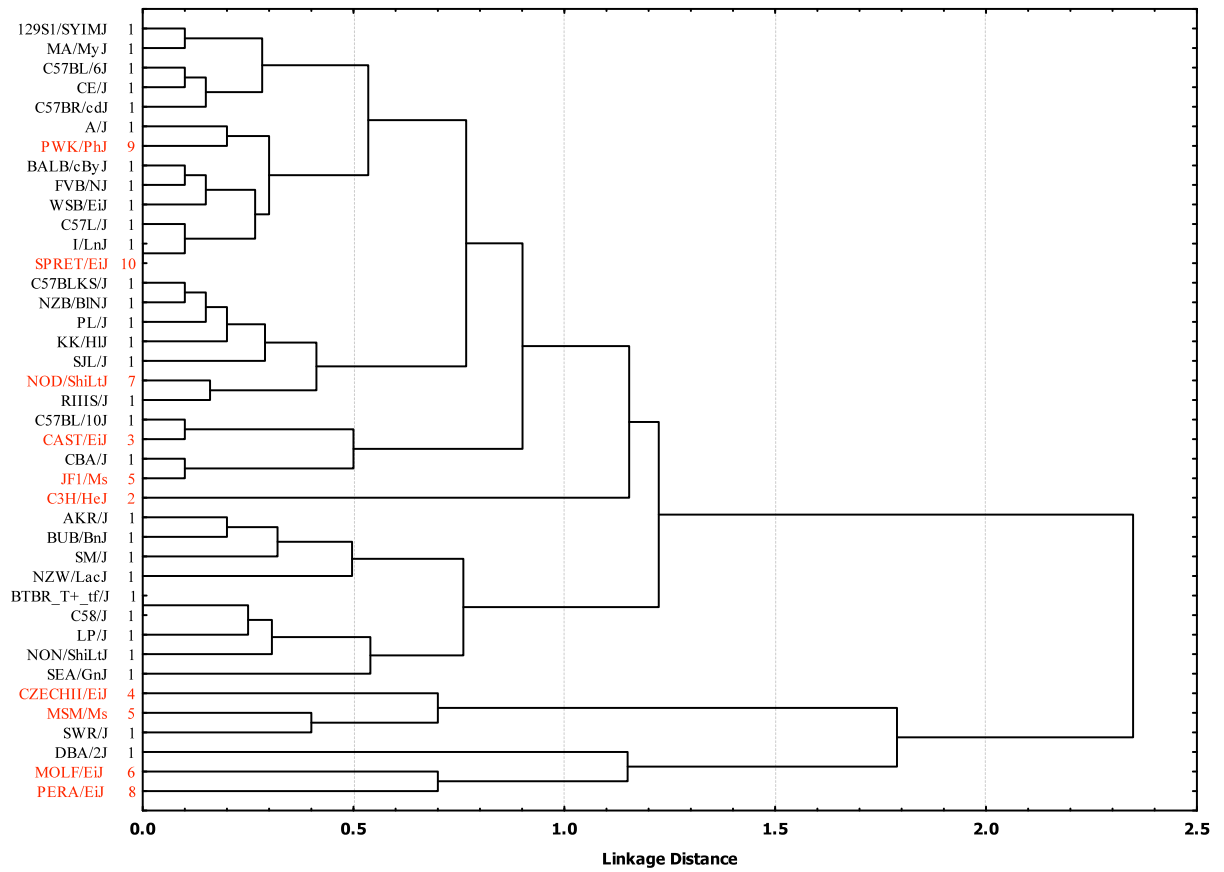

Dendrogram for 40 mouse strains based on BMD

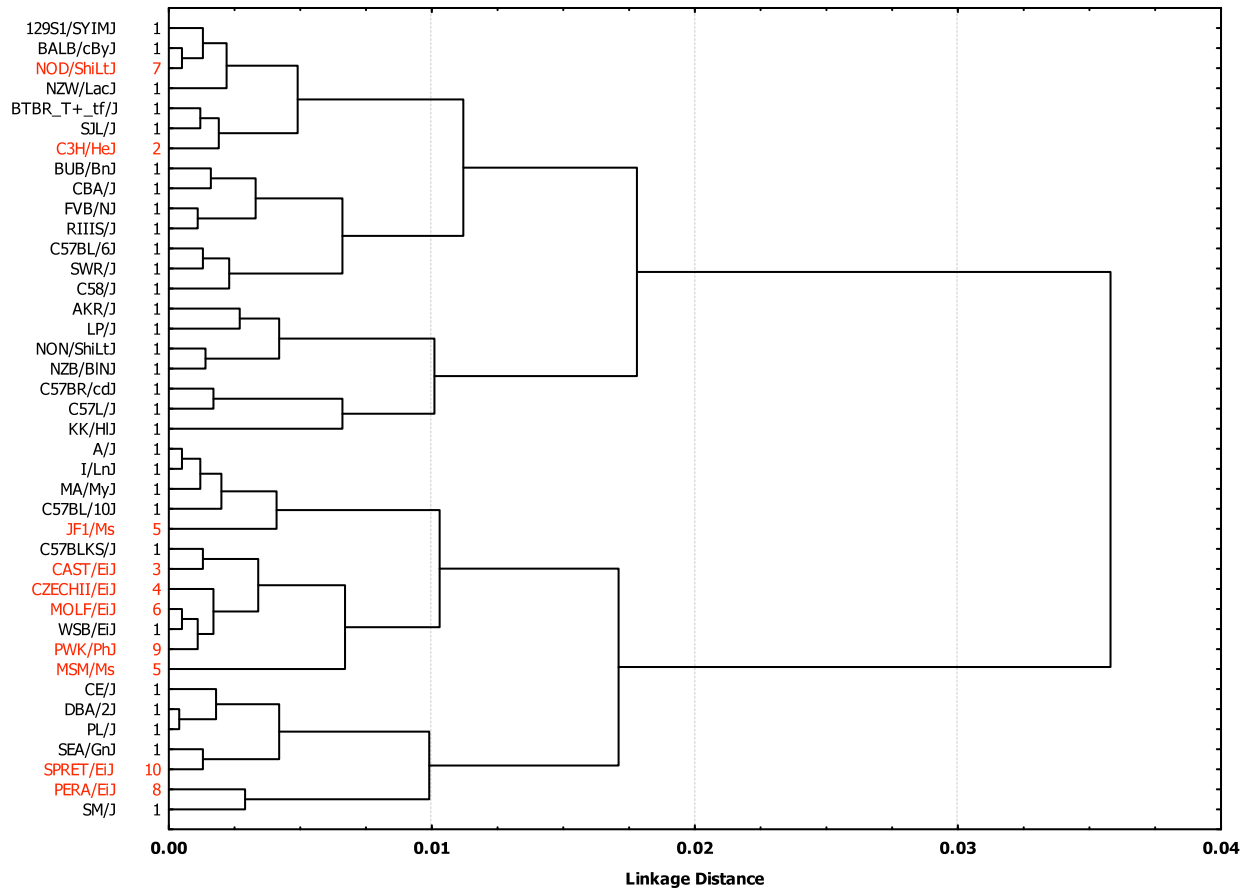

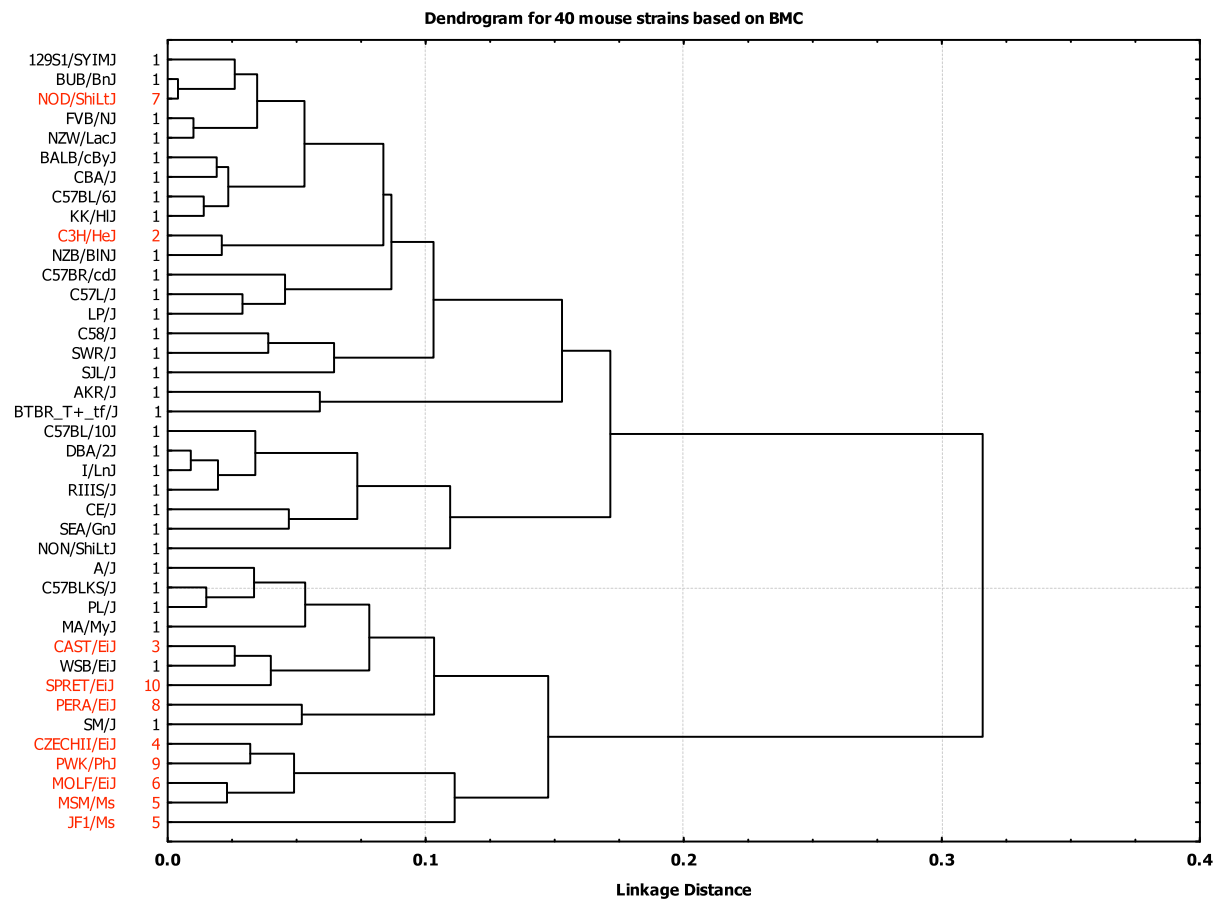

Supplement: Additional file 2 — Dendrogram for 40 mouse priority strains with haplotypes 1–10 (haplotype identifiers in parentheses) based on the prothrombin time (PT) and bone mineralization (BMD/C) values. Haplotypes different from those found in most of the commonly used strains are labeled in red. [file 1756-0500-1-125-S2.pdf]
